# The Conceptual Design of a Variable Camber Wing

**DOI:** 10.3390/biomimetics10060353

**Published:** 2025-06-01

**Authors:** Spencer Troy P. Cortez, Seksan Winyangkul, Suwin Sleesongsom

**Affiliations:** 1 Department of Aeronautical Engineering, International Academy of Aviation Industry, King Mongkut’s Institute of Technology Ladkrabang, Bangkok 10520, Thailand; 2 Department of Logistic Engineering and Management, Faculty of Industrial Technology, Chiang Rai Rajabhat University, Chiangrai 57100, Thailand

**Keywords:** variable camber wing, four-bar linkage, optimization technique, metaheuristics, TLBO

## Abstract

The variable camber wing (VCW) is a morphing wing design anticipated to enhance unmanned aerial vehicles’ (UAVs’) performance in flight through continuously changing shape. The performance of VCWs has been proven, but techniques for their integration, including aerodynamic analysis, mechanism synthesis, and structural tests, still lag in development at the conceptual design stage. Therefore, this research focuses on designing a variable camber wing, a key area for the advancement of morphing aircraft. Inspired by the high-lift capabilities of traditional aircraft devices but aiming for smoother airflow through continuous shape alteration, this research proposes a novel three-step design for a structurally integrated VCW. This approach begins with a critical aerodynamic analysis to determine wing shape adaptations across various flight conditions, followed by a mechanism synthesis phase to design a four-bar linkage that accurately approximates the desired trailing edge deflections by utilizing a variant of teaching–learning-based optimization. The objective is to minimize error between the intended and actual coupler link while adhering to design constraints for proper integration in the wing structure. Finally, structural analysis evaluates the skin’s ability to withstand operational loads and ensure the integrity of the VCW system. The design result demonstrates the success of this three-step approach to synthesizing a VCW mechanism that meets the defined aerodynamic (actual deflection of 9.1764°) and structural targets (maximum Von Mises stress of 81.5 MPa and maximum deflection of 0.073 m), paving the way for enhanced aircraft performance.

## 1. Introduction

A high-lift system is needed for general aircraft, including unmanned aerial vehicles (UAVs) with a fixed camber, in which aerodynamic performance under different flight conditions is limited due to separation flow caused by the high-lift mechanism. Variable camber wings (VCWs) have received increased attention in the aviation industry due their potential to improve aircraft performance through in-flight wing shape adaptations. The concept stems from the term “morphing wing”, which is an aircraft wing that can become smooth and change shape during flight. The first aircraft with morphing wings was developed by the Wright brothers, who took inspiration from birds in flight. At the time, the technology was called wing warping. The structure of the wing was made from a wooden frame, muslin fabric cover, and control surfaces. Wing transformation or wing morphing, inspired by bird wings, which can change shape continuously in flight, was expected to increase aerodynamic performance. Currently, morphing wing concepts are divided into camber variation or variable camber wings, lateral wing bending, and wing twisting. The morphing wing is at the forefront of aviation technology, contrasting with traditional aircraft wings. The design can enhance an ideal airfoil for a single flying situation: morphing wings are engineered to optimize the wing shape in accordance with specific flight conditions. They enhance fuel efficiency and overall aerodynamic performance by optimizing lift and drag characteristics for various phases of flight.

The advantages of morphing wings have been studied in comparison with traditional shapes, demonstrating their performance [1]. In the study of morphing wings, the concept can be classified into three broad groups, namely, in-plane morphing, airfoil morphing, and out-of-plane morphing [2]. A common conventional device for generating additional lift was inspired by the high-lift mechanism, which is an efficient means of generating additional lift for the main body wing. It is included in the group of camber changes. A simple type of camber change is a technique based on mechanism synthesis methodology, which can also be categorized as structure-based. The morphing material is important to the mechanism used to control shape changes. One example of a structure-based morphing camber wing is presented in [3]. The technique applied torque to the leading and trailing edges to change the shape of the airfoil through a small slit on the wing body. The resulting wing can change its leading and trailing edges. The two dimensions of the proposed adaptive variable camber wing concept include the self-rotation of the slicing rib into multiple parts. The slicing rib parts connect with a pivot to enable self-rotation [4]. A sliding trailing edge for UAVs with different sliding motions of the upper and lower skins was developed to attach to the ribs, which enables a sliding motion [5]. Performance was measured according to the lift-to-drag ratio in comparison with conventional high-lift devices, similar to [4], leading to a multiunit self-rotating rib [6] that can rotate around revolute joints. A corrugated VCW was embedded at the leading and trailing edges to generate smooth deflection [7]. In previous work [4,6], a variable camber mechanism (VCM) was developed based on a multi-slotted mechanism [8]. This concept was extended to the linkage morphing mechanism [9,10], which can be modeled in two- and three-dimensional simulations.

A previous work enhanced performance using camber morphing airfoils [11]. Part of the study involved using the nonlinear properties of the material to develop variable-structure models for more variable loading. This approach is a material-based variable camber change technique. It causes morphing wings to enhance the lift-to-drag ratio, making aerial vehicles more effective by allowing for continual geometric change. This discovery supported the potential advantages of VCWs. Later, it was extended to new mechanisms for VCWs. A general VCW includes a planar transmission mechanism that converts linear motions to the rotational and pitch wing. All motions are expected to enhance aerodynamic performance. This work expands the scope of variable camber concepts by providing real-world examples of morphing technologies in smaller-scale devices, which complements earlier research. Based on the expectations of VCWs, a comprehensive technique for optimizing the aerostructural wing shape that considers coupling between structural and aerodynamic nonlinearities was presented by the authors of [12]. The results indicated poor aerodynamic performance when structural flexibility was ignored, emphasizing the need for integrated strategies in VCW design. Furthermore, aeroelastic tailoring for aerospace applications is needed to explore how novel structural configurations can enhance aircraft performance while minimizing weight [13]. The results were confirmed with experimental and numerical investigations that revealed the significant impact of rib and spar configurations on the aeroelastic behavior of wings. By demonstrating the benefits of internal structural variations and additively manufactured lattice structures, this research highlights ongoing innovation within the field, emphasizing the critical role of structural optimization in the development of advanced VCWs. Together, these articles comprise an extensive body of literature that not only demonstrates the development of VCW design but also illustrates the significance of interdisciplinary methodologies used to overcome challenges linked to aerodynamic efficiency and structural integrity in modern aerospace engineering. Recent new research on VCWs has explored the use of mechanism synthesis approaches such as the scissor VCM [14] and variable camber trailing edge driving with Stephenson six-bar linkage [15]. The performance of these designs was proven using prototypes and wind tunnel testing [16,17]. The potential benefits of this mechanism for VCM design are a current topic of interest, causing research on variable camber wings using four-bar linkage (FBL) path synthesis to increase steadily. These new concepts and methods focus on improving aerodynamic efficiency through innovative mechanism designs. VCWs can enhance aerodynamic performance in different flight stages, resulting in benefits such as reduced fuel consumption and increased operational flexibility. It is challenging to add weight and mechanical complexity. However, FBL continues to be used in high-lift systems due to its durability and ease of maintenance compared to other types of actuators, such as electromechanical and thermo-electric actuators. If the VCW segment is too heavy, electro-actuators will cause control problems. In contrast, the four-bar mechanism has been proven capable of carrying a heavy structure. These factors can confirm the need for a reliable and cost-effective design to ensure the system’s feasibility in practical applications. Which mechanism has more than a thousand uses and is applied in daily life? Many machines utilize an FBL mechanism, which is applied in high-lift mechanisms, front wheel suspensions, oil wells, windshield wipers, etc. The design of the four-bar path and motion generation has significantly progressed due to the path-repairing technique, which simplifies the four-bar design [18]. In the context of VCWs, FBL offers an efficient way to translate rotational motion into controlled, complex movements required to adjust wing shapes dynamically. The design of such a mechanism involves path generation, function generation, and motion generation. Furthermore, FBL mechanisms provide an effective path synthesis approach to achieve the precise, reliable movements necessary for such wing adjustments.

Several innovative design concepts are being explored to realize the potential of variable camber technology. One approach involves flexible wing structures, which utilize materials or designs that can deform under controlled actuation. This can include smart materials, compliant mechanisms, or more general morphing wing concepts [19]. Another method focuses on segmented wing designs, in which the wing is divided into multiple independently actuated segments to alter the overall camber. Deployable camber devices represent another avenue, utilizing mechanisms that deploy or retract to modify the wing’s curvature, similar to but potentially more extensive than traditional flaps and slats [20]. Finally, variable-geometry airfoils are being investigated, which involves designing airfoils with internal mechanisms capable of changing the shape of the airfoil profile [21].

Furthermore, developing efficient, reliable, and lightweight actuation systems is crucial for controlling wing shape changes, with various actuation technologies being explored. Effective control strategies are also necessary to optimally manage the wing camber in response to flight parameters and control laws. Weight, complexity, and maintenance issues are some of the challenges associated with the system design and realistic integration of VCW systems into current or future aircraft designs. Finally, advancements in manufacturing and material science are vital to enabling the fabrication of complex and dynamically adaptable wing structures [22].

Given the literature review, this research proposes a three-step approach to designing a variable camber wing for a UAV, beginning with aerodynamic analysis to determine the target wing shapes that will increase performance under various flight conditions. Subsequently, a mechanism synthesis stage verifies that the linkage design can fulfill the mechanical requirements for smooth operation. The key is to minimize the summed squared error (SSE) between the predefined target points and the actual points traced by the coupler link. Finally, the ability of the VCW’s skin to handle the deflection load and aerodynamics when working as a morphing wing is tested.

## 2. Research Methodology

### 2.1. Aerodynamic Theory of VCWs

The vortex lattice method (VLM) was employed in the aerodynamic analysis in this study to efficiently evaluate the aerodynamic performance of the variable camber wing across various camber configurations. The VLM is a numerical technique that models the wing as a series of discrete panels, each containing bound vortices. By enforcing the boundary condition that the flow must be tangent to the wing surface, the strength of these vortices is determined, enabling the calculation of lift and induced drag [23]. The technique was developed for steady, quasi-steady, and unsteady conditions for multi-disciplinary design optimization problems [24] and is particularly well suited to analyzing the overall lift distribution and induced drag characteristics of wings, offering a good balance between accuracy and computational cost for initial design studies. In this research, the VLM was used to analyze the lift and induced drag characteristics of a VCW at different deflection angles. For each deflection, the wing geometry was discretized into a lattice of quadrilateral panels, and the resulting system of equations was solved to obtain the vortex strengths. Although the VLM offers computational efficiency in initial design studies, it is essential to acknowledge its limitations, particularly its simplified treatment of viscous effects, which can become significant in certain flow regimes. In this research, an NACA 2412 airfoil was used as a VCW shape in two dimensions. Its aerodynamic performance following shape changes was analyzed using XFOIL 6.99 at different trailing edge deflections [0–10°], with an interval of 2°. In this study, the Mach number was set at 0.2, the Reynolds number was set at 1 × 10^6^, and the angle of attack was set at 3°. The testing conditions matched those in [25,26]. The variable camber change was affected by lift, drag, and the lift-to-drag ratio. Furthermore, the resulting data from XFOIL, presented in Table 1, provided the target points of the tailing edge. The next section describes efforts to replicate its actual points via four-bar mechanism synthesis.

### 2.2. Four-Bar Linkage Synthesis for VCWs

**Figure 1:**
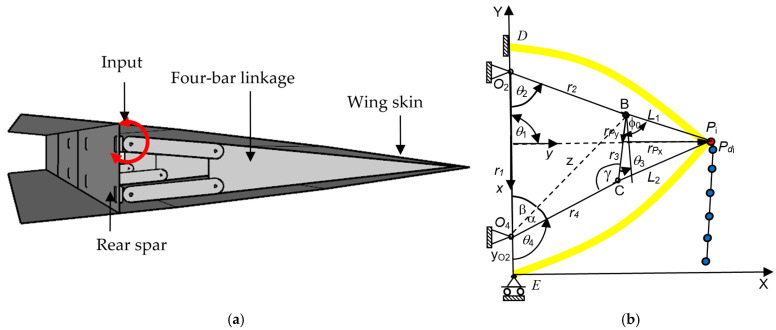
(**a**) The variable camber mechanism. (**b**) A kinematic diagram of a VCM.

A four-bar linkage is a simple yet versatile mechanical system that consists of four rigid links connected by revolute joints. In the case of variable camber wings, the four-bar linkage allows for controlled, continuous deformation of the wing profile to adapt to different flight conditions, as shown in Figure 1a. This is critical in enabling wings to efficiently transition between different aerodynamic profiles during flight. In variable camber wing applications, it is critical to design the four-bar linkage to be a double rocker. The path-generation problem for a VCM is presented below.

The linkage only needs one input applied at link 2, as shown in Figure 1b. The trigonometric is used to analyze the position of the four-bar linkage. The relationship is in the form of the linkage lengths *r*_1_, *r*_2_, *r*_3_, *r*_4_, *θ*_1_, *θ*_2_, *x_O_*_2_, *y_O_*_2_, *L*_1_, and *L*_2_, which was proven in previous work [18,27]. The coupler point (*P*) in the global coordinate in Figure 1b is expressed as

(1) $$\begin{array}{l} x_{P}=x_{O2}+r_{2}\cos(\theta_{2}+\theta_{1})+L_{1}\cos(\varphi_{0}+\theta_{3}+\theta_{1})\\ y_{P}=y_{O2}+r_{2}\sin(\theta_{2}+\theta_{1})+L_{1}\sin(\varphi_{0}+\theta_{3}+\theta_{1}) \end{array}$$

where *x_O_*_2_ and *y_O_*_2_ are the global coordinate positions of *O*_2_ [18]. The angle *θ*_1_ is a different angle between the local coordinate *x*-*y* and the global coordinate *X*-*Y*. The angle *ϕ*_0_ can be obtained by considering the coupler link *BCP* using the cosine law, which is expressed as

(2) $$\varphi_{0}=\cos^{-1}\left[\frac{L_{1}^{2}+r_{3}^{2}-L_{2}^{2}}{2L_{1}r_{3}}\right]$$

At the input crank angle (*θ*_2_), the values of angles *θ*_3_, *θ*_4_, and *γ* for the link lengths *r*_1_, *r*_2_, *r*_3_, and *r*_4_ are determined as follows:

(3) $$z^{2}=r_{1}^{2}+r_{2}^{2}-2r_{1}r_{2}\cos\theta_{2},\;z^{2}=r_{3}^{2}+r_{4}^{2}-2r_{3}r_{4}\cos\gamma$$

(4) $$\gamma =\cos^{-1}\left[\frac{r_{3}^{2}+r_{4}^{2}-r_{1}^{2}-r_{2}^{2}+2r_{1}r_{2}\cos\theta_{2}}{2r_{3}r_{4}}\right]$$

(5) $$\gamma =\cos^{-1}\left[\frac{r_{3}^{2}+r_{4}^{2}-z^{2}}{2r_{3}r_{4}}\right],\;\alpha =\cos^{-1}\left[\frac{z^{2}-r_{3}^{2}+r_{4}^{2}}{2zr_{4}}\right]$$

(6) $$\beta =\cos^{-1}\left[\frac{z^{2}+r_{1}^{2}-r_{2}^{2}}{2zr_{1}}\right],\;\theta_{3}=\pi -(\alpha +\beta +\gamma )$$

(7) $$\theta_{4}=\pi -(\alpha +\beta )$$

### 2.3. VCW Mechanism Synthesis

In variable camber wing applications, it is critical to design the four-bar linkage so that it enables the desired wing deformation profile while adhering to the constraints imposed by Grashof’s Criterion. In this case, a double-rocker configuration is required, in which two links partially rotate in a circle while the tailing edge follows a specific path set by the coupler link. The specific path is determined by the simulation of the previous target points via XFOIL for a deflection angle from [0–10°] at an interval of 2°. The objective function is the position error between the target points *P_d_*(*x_d_*, *y_d_*) and the actual points *P*(*x_p_*, *y_p_*), as presented in Figure 1b. The design variables in this problem included *r_1_*, *r_2_*, *r_3_*, *r_4_*, *r_Px_*, and *r_Py_*. The input set of *θ*_2_*^i^* values was also set as the design variables. Then, the optimization problem without prescribed timing is written as

(8) $$\mathrm{Min}\;f(x)=\sum_{i=1}^{N}[{\left(x_{d,i}-x_{p,i}\right)}^{2}+{\left(y_{d,i}-y_{p,i}\right)}^{2}]$$

Subject to

min(*r*_1_, *r*_2_, *r*_3_, *r*_4_) = coupler(*r*_3_) (9)

2 min(*r*_1_, *r*_2_, *r*_3_, *r*_4_) + 2 max(*r*_1_, *r*_2_, *r*_3_, *r*_4_) < (*r*_1_ + *r*_2_ + *r*_3_ + *r*_4_) (10)

(11) $$\theta_{2}^{1}<\theta_{2}^{2}\;\;\ldots \;\;<\theta_{2}^{N}\;$$

*x*_l_ ≤ *x* ≤ *x*_u_. (12)

where *x* = {*r*_1_, *r*_2_, *r*_3_, *r*_4_, *r_Px_*, *r_Py_*, *θ*_2_*^i^* }*^T^*, *N* = 6 is the number of points on the prescribed or target path due to trailing edge deflection at different angles, as shown in Table 1, and *x*_l_ and *x*_u_ are the lower and upper bounds of the design vector *x*, respectively. By applying the proper constraints, this simulation can be used to represent the behavior of the VCW. The additional constraints used for defining the workspace include the coordinates of *O*_2_ (*x*_O2_, *y*_O2_) and the angle of link 1 (a frame) (*θ*_1_):

*θ_1_* = −90^°^, *x*_O2_ = 0, *y*_O2_ = 0.07 m (*O*_2_ and *O*_4_ are attacked at the rear spar) (13)

The limits of the design variables are as follows:

0.05 ≤ *r_1_*, *r_2_*, *r_3_*, *r_4_* ≤ 1.5 m (14)

0.05 ≤ *r_px_*, *r_py_* ≤ 1.5 m (15)

0 ≤ *θ*_2_*^i^* ≤ 30° (16)

### 2.4. Optimization Technique

While Grashof’s Criterion provides a fundamental guideline for determining the motion capabilities of a four-bar linkage, additional optimization techniques are required to fine-tune the linkage dimensions and ensure that the system performs optimally within aerodynamic and design space constraints.

TLBO-DA optimization was selected due to its efficiency in handling constrained optimization problems and ability to converge with fewer hyperparameters compared to genetic algorithms [18]. This research employed Teaching–Learning-Based Optimization with Diversity Archive (TLBO-DA) to solve the optimization problem. This algorithm mimics a classroom teaching process as follows: in the teaching phase, the teacher (the best solution) updates the students (the remaining solutions) based on their relative performance. In the learning phase, the students interact and learn from one another to refine their solutions. A diversity archive maintains diverse solutions to avoid premature convergence. The algorithms were coded in MATLAB 2024a commercial software with the following initial settings: the population size was set at *n_P_* = 100, the number of iterations was set at 500, and the number of runs of the simulation was set at 30. Algorithm 1 is presented below.
**Algorithm 1.** ATLBO-DA AlgorithmInput: maximum number of generations (*n_it_*), population size (*n_P_*)
Output: x^best^, *f*^best^ Initialization: Step 0.1 Generate *n_p_* initial students {x*^i^*} and perform function evaluations {*f^i^*}. Step 0.2 Initiate four 1 × 2 matrices, *TRR_Success*, *TRR_Fail*, *LRR_Success* and *LRR_Fail,* all of which elements are set to be ones. Main procedure Step 1 While (the termination conditions are not met) do {*Teacher Phase*} Step 2 Calculate the mean position of solutions {x*^i^*} written as M*_avg_*. Step 3 Calculate the probabilities of selecting the intervals for *T_RR_*: $PTRR_{j}=\frac{TRR\_Success_{j}}{TRR\_Success_{j}+TRR\_Fail_{j}}$. Step 4 For *i* = 1 to *n_P_* Step 4.1 Perform roulette wheel selection with *PTRR_j_*. Step 4.1.1 If *j* = 1 is selected, *T_RR_* = 0.4 + 0.1 *rand* is sampled. Step 4.1.2 Else, if *j* = 2 is selected, *T_RR_* = 0.5 + 0.1 *rand* is sampled. Step 4.2 Generate *P_r_* = *rand* and select a teacher. Step 4.2.1 If *P_r_* ≤ *T_RR_*, set the best solution as a teacher M_best_. Step 4.2.2 Else, if *P_r_* > *T_RR_*, randomly select a solution in A*_D_* and set it as a teacher M_best_. Step 4.3 Create x*^i^*_*new*_ using following formular and perform function evaluation. x_*new*_ = x_*old*_ + *Difference*_*Mean* where *Difference*_*Mean_i_* = *r_i_* (M*_i_*, _best_ − *T_f_*M*_i_*_,avg_), and *T_f_* = round(1 + *r*_i_). Step 4.3.1 If x*^i^*_*new*_ is better than x*^i^*, add 1 point to the *j*-th element of *TRR_Success*. Step 4.3.2 Else, add 1 point to the *j*-th element of *TRR_Fail*. Step 5 Replace {x*^i^*} by *n_P_* best solutions from {x*^i^*}∪{x*^i^*_*new*_}. {*Learning Phase*} Step 6 Calculate the probabilities of selecting the intervals for *L_RR_* similar to that for *T_RR_* in step 3. Step 7 For *i* = 1 to *n_P_* Step 7.1 Perform roulette wheel selection with *PLRR_j_*. Step 7.1.1 If *j* = 1 is selected, *L_RR_* = 0.4 + 0.1 *rand* is sampled. Step 7.1.2 Else, if *j* = 2 is selected, *T_RR_* = 0.5 + 0.1 *rand* is sampled. Step 7.2 Generate *P_r_* = *rand*. Step 7.2.1 If *P_r_* ≤ *L_RR_*, create x*^i^*_*new*_ using two-student learning and perform function evaluation. Step 7.2.2 Else, create x*^i^*_*new*_ using three-student learning and perform function evaluation. Step 7.3 Update *LRR_Success* and *LRR_Fail*. Step 7.3.1 If x*^i^*_*new*_ is better than x*^i^*, add 1 point to the *j*-th element of *LRR_Success*. Step 7.3.2 Else, add 1 point to the *j*-th element of *LRR_Fail*. Step 8 Replace {x*^i^*} by *n_P_* best solutions from {x*^i^*}∪{x*^i^*_*new*_}. Calculate the objective function value of x*^i^*_*new*_. Step 9 Update the diversity archive with the non-dominated solutions obtained from {x*^i^*}_*old*_∪{x*^i^*}_*new*_. Step 10 End While

### 2.5. Structural Strength

#### 2.5.1. Response Surface

An aerodynamic force applied to the VCW structure can be modeled using a regression technique with the response surface method (RSM). The regression function of the lift force from the VLM, which depends on the position in chord and span, can be modeled. The general form of two independent variables of second-order regression can be expressed as follows:

(17) $$y=\beta_{0}+\beta_{1}x_{1}+\beta_{2}x_{2}+\beta_{11}x_{1}^{2}+\beta_{22}x_{2}^{2}+\beta_{12}x_{1}x_{2}+\varepsilon$$

where *y* is the output; *x*_i_ is the *k* independent variable; and *β_j_*, *j* = 0, 1, …, *k* are regression coefficients. The regression coefficients are approximated using VLM results *y*_i_, *i =* 1, …, *n,* where *n* is the number of panels and *n* > *k.* In this case, this is the lift force. The use of the least squares technique system equation in (17) can change the equation to matrix form:

(18) y=Xβ+ε

where output y is a vector *n* × 1, X is a matrix *n* × *p* (*p* = *k* + 1), β is a vector of the regression coefficient *p* × 1, and ε is a vector of the residual error *n* × 1. Then, the regression coefficient can be calculated as follows:

(19) $$b={\left(X^{\prime }X\right)}^{-1}X^{\prime }y$$

Good model fitting regarding lift forces and the response surface method requires statistical analysis to ensure the forces are transferred precisely to the finite element model. In this study, the fitting quality of the model is considered in the form of the determination coefficient (*R*^2^) and adjusted determination coefficient (*R*^2^*_adj_*). *R*^2^ and *R*^2^*_adj_* are calculated as follows:

(20) $$R^{2}=1-\frac{SS_{E}}{SS_{T}}$$

(21) $$R_{adj}^{2}=1-\frac{SS_{E}/(n-p)}{SS_{T}(n-1)}=1-\frac{n-1}{n-p}(1-R^{2})$$

where the residual of sum squares $SS_{E}=y^{\prime }y-b^{\prime }X^{\prime }y$, and the total of sum squares $SS_{T}=y^{\prime }y-\frac{{\left(\sum_{i=1}^{n}y_{i}\right)}^{2}}{n}$. The determination coefficients are in [0, 1] and higher than 0.8, representing a good fit [28].

#### 2.5.2. Finite Element Analysis

The structural strength of the variable camber wing was analyzed using both deflection and Von Mises stress criteria. Deflection provides a fundamental understanding of how the wing deforms under aerodynamic loading, ensuring that the displacement remains within acceptable limits to maintain aerodynamic performance. The deflection and stress of the VCW structure were computed using the finite element method (FEM) as follows.

The static equation for aircraft structure can be presented as

(22) [K]u=F

where [*K*] is the stiffness matrix of a structure, u is the nodal displacement vector, and F is the vector of external forces.

The finite element technique divides the aircraft structure into finite elements to determine the global stiffness *K*. To solve the static analysis in (22), the force applied to the structure is derived using the VLM, as mentioned in the previous section where $L=\rho U_{\infty }\Gamma$. $\rho_{air}$ is the mass density of the air, Γ is the vortex strength, and $U_{\infty }$ is the aircraft speed. The lift formula is accorded using the Kutta–Joukowsky theorem.

The aerodynamic load acting on the aircraft structure (22) is determined using the regression technique, as noted in the previous section. The displacement vector can then be solved. In a linear analysis of the structure, the stresses inside the aircraft structure can be solved. In this study, the equivalent stress in the form of the Von Mises equivalent stress is expressed as follows:

(23) $$\sigma ={\left(\sigma_{11}^{2}+\sigma_{22}^{2}-\sigma_{11}\sigma_{22}+3\sigma_{12}^{2}\right)}^{1/2}$$

where $\sigma_{ij}$ is the stress component.

Von Mises stress is a widely used failure criterion in structural analysis, as it accounts for the combined effects of different stress components [29]. The structural model was subjected to aerodynamic loads derived from the vortex lattice method (VLM), which were then applied as nodal forces in the finite element model. The maximum Von Mises stress was evaluated to determine if the material would yield under the given loading conditions. The stress distribution results revealed that the VCW structure remained within the material’s yield strength, ensuring structural integrity during operation.

These findings align with previous studies on morphing wing structures and emphasize the importance of optimizing material properties and structural reinforcements to achieve lightweight and durable morphing designs [30]. Further research on compliant mechanisms and advanced composite materials has shown the potential to reduce structural weight while maintaining strength. Additionally, recent studies highlight the importance of aeroelastic tailoring in morphing wings to mitigate flutter and enhance overall stability [29].

## 3. Results and Discussion

**Figure 2:**
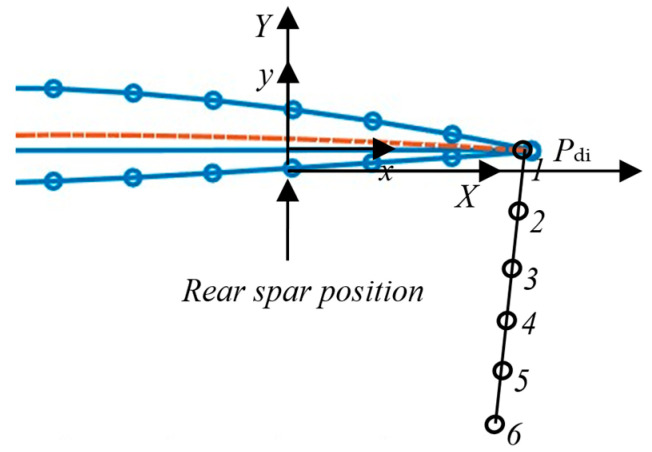
The design space of the VCW mechanism at the trailing edge.

This section outlines the key outcomes of this research on designing a four-bar linkage mechanism for a variable camber wing. The proposed technique in this study can be separated into three main stages. First, aerodynamic analysis (Section 2.1) is used to determine the ideal tailing edge shape adjustments needed for improved flight performance. Using XFOIL and focusing on an NACA 2412 airfoil, this study simulated how different trailing edge deflections affected lift, drag, and the lift-to-drag ratio. The resulting aerodynamic performance data for difference tailing edge deflection angles [0–10°] with an interval of 2° are presented in Table 1, which follows the coordinates shown in Figure 2. The experiments provided the target points that were used as target points for the four-bar mechanism synthesis in Section 3.2.

**Figure 3:**
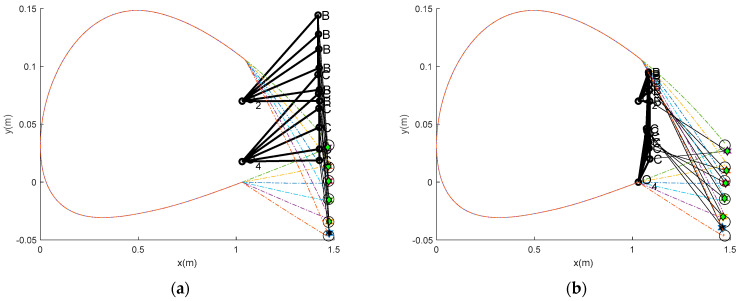
(**a**) Worst VCM. (**b**) Best VCW mechanism.

Next, Section 3.2 details the mechanism synthesis phase. Here, the goal was to design a four-bar linkage that could accurately achieve the target points of the tailing edge. This research employed an optimization technique using the TLBO-DA algorithm to explore various linkage configurations. Figure 3 shows the best and worst results from 30 runs. Table 2 provides the specific dimensions and descriptive statistics. The results indicate successful synthesis, with the double-rocker identified as the most effective design, meeting the necessary kinematic requirements.

This research also considered crucial structural aspects. The design had to ensure that the variable camber skin could withstand aerodynamic forces acting on the wing without compromising its structural integrity. In essence, this research summarizes a journey through these three stages—from defining the aerodynamic goals to designing a mechanism capable of achieving them—while also considering the structural robustness required for a functional variable camber wing system.

### 3.1. The Desired Aerodynamic Performance of the Wing

The aerodynamic performance of a variable camber wing depends heavily on its ability to adjust its camber in response to changing flight conditions. In addition, this ability is necessary for handling preloads and carrying additional loads in UAV missions. The use of a four-bar linkage provides a mechanically simple and efficient way to achieve these adjustments. However, the dimensions of the linkage must ensure that the range of motion achieves the desired wing deformation targets without exceeding the design space. An aerodynamic analysis of trailing edge deflection with different angles was conducted using the vortex lattice method in the free software XFOIL 6.99 [31]. The aerodynamic performance is expected to increase the lift and lift-to-drag ratio and handle drag. Following this, the NACA 2412 airfoil shape was selected. This airfoil is popular because it offers a good balance of lift and drag, making it efficient for small and moderate-speed UAVs. Its curved shape helps generate lift at lower speeds, and it has a predictable and stable performance. It is also easy to work with and has gentle stall characteristics, increasing its reliability and safety, especially in UAVs and light aircraft. Overall, it is a well-rounded design that works in various flying conditions. The Mach number was set at 0.2, the Reynolds number was set at 1 × 10^6^, and the angle of attack was set at 3° in this study.

The target points at the desired deflection angles of the tailing edge are shown in Table 1, while the local coordinate, global coordinate, and offset distance are shown in Figure 2. The aerodynamic performance includes the lift, drag, and lift-to-drag ratio coefficients, which were tested at different deflection angles [0–10°] and intervals of 2°. The data are presented in Table 1. First, the coefficient of lift in the present study under the test conditions (an angle of attack of 3° without a deflection angle or a deflection angle of 0°) is in accordance with the data in [25,26] at 0.610, while the present coefficient of lift is 0.6034. This study was used to confirm the validation step. Given the results, the desired deflection angles can enhance the lift performance and lift-to-drag ratio to handle drag, which is the aim of the variable camber wing. The VCW starts morphing with a lift coefficient of 0.6034 (0°) and increases to the highest lift at 1.1787 (10°), while the lift-to-drag ratio initially starts at 92.83 (0°) and then increases to 115.16 (4°), finally reducing to 85.41 (10°). These results are consistent with the results reported in a study testing the NACA 2412 airfoil at different deflection angles [−3°, 6°] with an interval of 1° for both an experiment [25] and simulation [26]. Furthermore, the results show that the coefficient of the lift-to-drag ratio can increase to over 100, which is in accordance with [25,26].

### 3.2. Mechanism Synthesis

The results of the four-bar linkage synthesis design for the VCM are shown in Figure 3 and Table 2. The successful run number in the table is the optimization run, during which a feasible linkage was found, ranging from 16 to 30. The unsuccessful run indicates the worst performance. The results also include the mean objective function values from the successful runs (mean), the successful run result with the highest error (max), the best feasible result (min), and the standard deviation (std). The descriptive statistics (mean, max, min, and std) show the performance of our proposed technique, which provides a unique solution to this design problem with a narrow standard deviation and the remaining statistics. Figure 3a shows one of the worst mechanisms, while Figure 3b shows the best mechanism (min). The figures were created after 30 runs, including the worst and best runs and their descriptive statistics. This means that the worst result is not included in the design space, while the best result is. The best result yielded an error of 0.000631 in counterclockwise rotation, which is higher than in the worst case (0.00005546 in clockwise rotation). Figure 3b shows that the best result outperforms the worst case because the VCM achieved the best fit with the trailing edge deflection. The dimensions of the link length in Table 2 show that the best results were found for the double-rocker type because the shortest link (*r*_3_) is opposite to the ground (*r*_1_). In contrast, the worst were obtained for the changed point type. This implies that Grashof’s double-rocker can be designed to meet the requirements. Given the results, the crank sequence of the best and worst results shows that the technique can perform well in tracking its crank sequence. The technique (the path-repairing technique) and the power of the optimizer (TLBO-DA) used in this study were sourced from a previous work [18]. The results confirm that this design satisfies Grashof’s Criterion and ensures that the linkage can achieve the continuous motion required for smooth camber transitions. Furthermore, the results confirm that the VCW can morph efficiently into different aerodynamic profiles during flight, as presented in the next section.

### 3.3. Actual Aerodynamic Performance and Structural Testing

To understand the structural behavior of a variable camber wing under a load, it is crucial to determine the actual aerodynamic performance and displacement of the trailing edge as the camber changes. Based on the desired positions outlined in Table 1, the corresponding displacements were calculated from the initial reference point. As illustrated in Figure 3b, the initial position of a specific point on the trailing edge is defined as (1.472, 0.0318) in the local coordinate system, which is denoted as "O". This serves as the reference for measuring subsequent displacements. For each target deflection angle (δ) listed in Table 3, the vertical displacement (Δy) of this trailing edge point was calculated, as shown in column 4. The actual deflected angle of the VCW can also be calculated as presented in column 3, and the aerodynamic performances of the actual VCW are presented in the last three columns. All comparative aerodynamic performance values are presented in the same column. The percentage differences confirm the ability of the optimum four-bar linkage to serve as a VCW mechanism. The largest differences occur with the initial movement and at the end of the movement, indicating a need for careful control at these points. In particular, the coefficient of lift at the target points 1–2 and 6 demonstrates the special “fast in, slow out” characteristic of the VCW. This characteristic can generate a higher lift than expected at the starting deflection and generates a lower lift until the end deflection. The largest deflected angle occurred at the desired angle (10°), so each displacement was calculated depending on this angle, as shown in column 4.

#### 3.3.1. Material Properties

The wing is made of aluminum, and its properties are outlined below (Table 4).

#### 3.3.2. Stress of VCW Trailing Edge

**Figure 4:**
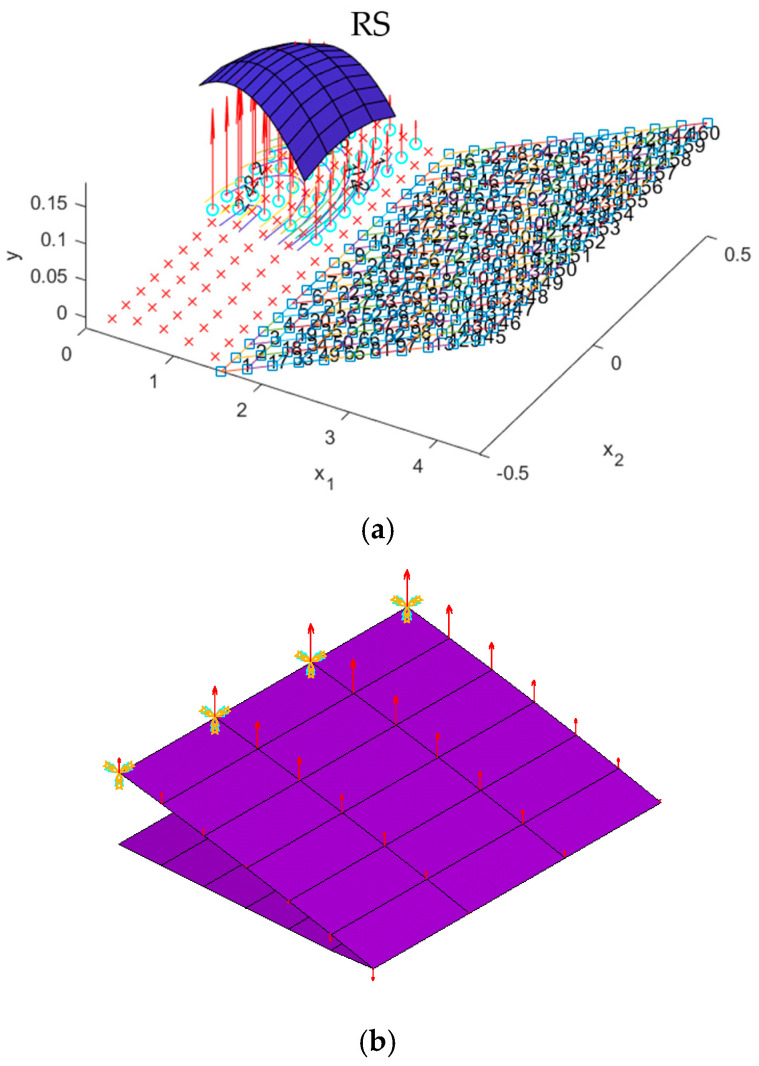
(**a**) The response surface (blue) of the lift force on the wing section at a certain angle of attack. (**b**) The lifting force on FEA nodes.

The stress and displacement results for the variable camber wing were obtained by setting the fixed-boundary condition at the root of the upper skin. The root of the bottom skin was only constrained in the *y* coordinate, as shown in Figure 1, and the displacement was applied due to the desired deflection angle and aerodynamic load in the FEA model. Aerodynamic lifting can be transferred via the VLM at the vertex points to FEA nodes in the form of the response surface technique (Section 2.5.1). The VLM was used to simulate the UAV’s straight wing section, which is 0.5 m in span and 1.472 m in chord length, with a 2412 airfoil profile. The model was placed with quadrilateral panels 5 and 8 chordwise and spanwise for the wing body, respectively, while its wake was 10 boxes chordwise, as shown in Figure 4a. The angle of attack in each simulation was used in the same manner as the angle of deflection of the tailing edge. The aircraft’s speed or free stream velocity in this simulation was 40 m/s. The quality of a regression function can be measured in the form of *R*^2^ and *R*^2^*_adj_*. The *R*^2^ and *R*^2^*_adj_* of the present regression function were 0.9319 and 0.9219, respectively. The RS (Figure 4a) and aerodynamic load on the FEA (Figure 4b) shape aligned with aerodynamic theory in terms of its elliptical shape [23], meaning that the lift force function can be used to generate the lifting force applied to the FEA model with good precision. The response surface (RS) is presented in Figure 4a.

**Figure 5:**
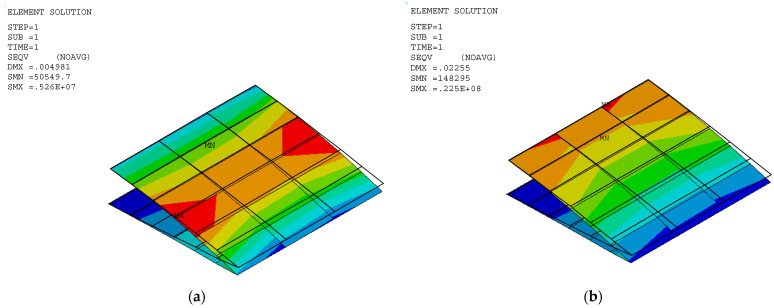
Stress distribution in a VCW at deflection angles of (**a**) 0° and (**b**) 2°.

**Figure 6:**
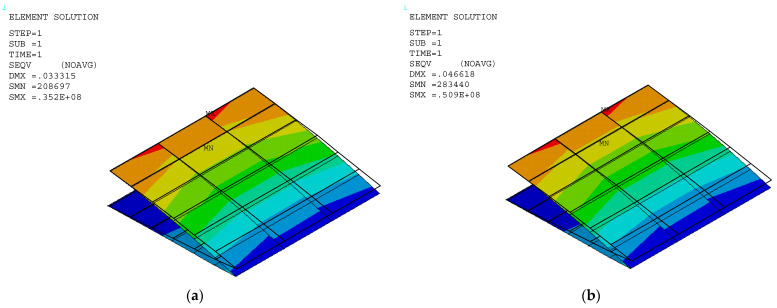
Stress distribution in a VCW at deflection angles of (**a**) 4° and (**b**) 6°.

**Figure 7:**
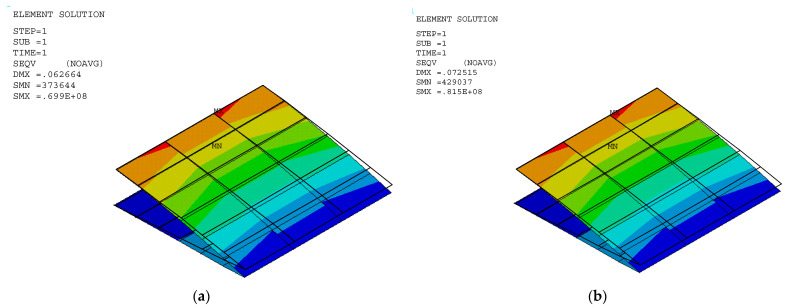
Stress distribution in a VCW at deflection angles of (**a**) 8° and (**b**) 10°.

**Figure 8:**
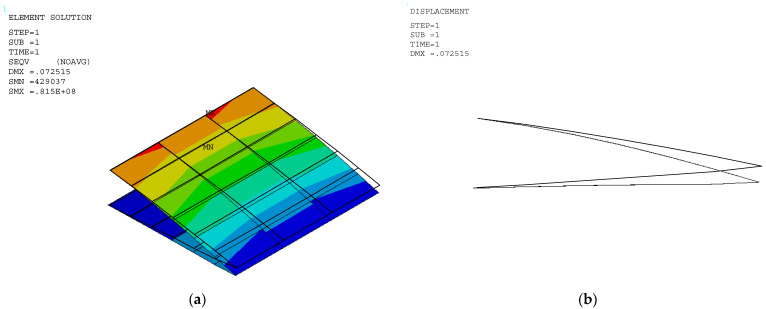
(**a**) Stress distribution in a VCW at deflection angle = 10° (**b**) deflection shape.

The stress distribution and structural flexibility are shown below in Table 5, and the stress and displacement analysis is shown in Figure 5, Figure 6 and Figure 7. The maximum Von Mises stress at 10° deflection was 81.5 MPa, which is considerably lower than the yield stress. The actual displacement values matched the desired displacement closely, confirming that the wing deformed smoothly as expected without excessive structural strain or uncertain aerodynamic performance. This reduction in stress suggests that the released bottom root attachment did not cause localized stress concentration, and local stress was minimized, leading to a more efficient load transfer. Additionally, since stress remained below the yield stress, the risk of plastic deformation was significantly reduced, improving the overall durability and reliability of the structure. These findings highlight the benefits of implementing greater structural flexibility in VCW design, which can ensure optimal performance without compromising material integrity. The wing deformation is as smooth as possible, as shown in Figure 8.

## 4. Conclusions

In conclusion, this study presents a three-step approach to designing a variable camber wing for a UAV using a four-bar linkage, building upon previous work [18]. This research successfully demonstrates the application of four-bar mechanism design principles to VCW path generation, identifying the double-rocker as the optimal configuration for smooth camber transitions. The path-repairing technique ensures continuous motion for efficient morphing, which can bring the VCW shape close to the desired aerodynamics of [0.722–9.714°]. The VCW passed a structural test, with the design limiting yield stress. Furthermore, structural analysis revealed that boundary condition placement significantly impacts the stress distribution under aerodynamic loads, emphasizing the need for careful structural considerations in future VCW designs.

In future work on VCW design, reliability-based design is necessary to suppress unwanted uncertainty due to loads and material properties.

## Figures and Tables

**Table 1:** Desired position and deflected angle of a variable camber wing.

Target Point	Position (m)	Angle (*δ*_i_, °)	*C_L_*	*C_D_*	*C_L_/C_D_*
1	(0.4416, 0.0318)	0	0.6034	0.0065	92.83
2	(0.4416, −0.0337)	2	0.7460	0.0069	108.12
3	(0.4413, −0.0626)	4	0.8752	0.0076	115.16
4	(0.4414, −0.0783)	6	1.0026	0.0093	107.81
5	(0.4415, −0.0980)	8	1.1008	0.0111	99.18
6	(0.4414, −0.1099)	10	1.1787	0.0138	85.41

**Table 2:** The design results of the VCM and descriptive statistics.

Parameter	Best	Worst
*r*_1_	0.0700	0.0522
*r*_2_	0.0579	0.3947
*r*_3_	0.0500	0.0514
*r*_4_	0.0621	0.3947
*r_Px_*	0.0500	0.1141
*r_Py_*	0.3957	0.0500
$\theta_{2}^{1}$	0	10.8650
$\theta_{2}^{2}$	9.2592	8.4342
$\theta_{2}^{3}$	14.1952	6.5619
$\theta_{2}^{4}$	19.1367	4.1709
$\theta_{2}^{5}$	23.6499	1.4421
$\theta_{2}^{6}$	25.7118	0.0209
min	0.000631	0.00005546
mean	0.000631	
max	0.000633	
std	5.67396 × 10^−7^	
Successful run	16	

**Table 3:** Corresponding displacements, actual aerodynamic performance, and actual deflection angles.

Target Point	Desired Angle (*δ_i_*°)	Actual Angle (*δ_aci_*°)	Displacement (Δy, m)	*C_L_* (% *Difference*)	*C_D_* (% *Difference*)	*C_L_*/*C_D_* (% *Difference*)
1	0	0.7220	0.005	0.6720 (**11.3689**)	0.0066 (**1.5385**)	101.3575 (**9.1861**)
2	2	2.7971	0.022	0.7967 (**6.7962**)	0.0072 (**4.3478**)	110.9610 (**2.6276**)
3	4	4.0892	0.033	0.8810 (**0.6627**)	0.0077 (**1.3158**)	114.7135 (**0.3877**)
4	6	5.7725	0.046	0.9887 (**1.3864**)	0.0092 (**1.0753**)	107.8190 (**0.0083**)
5	8	7.8541	0.062	1.0945 (**0.5723**)	0.0109 (**1.8018**)	100.3208 (**1.1502**)
6	10	9.1764	0.07	1.1487 (**2.5452**)	0.0125 (**9.4203**)	91.8225 (**7.5079**)

**Table 4:** Material properties.

Property	Value	Unit
Young’s modulus (E)	70 × 10^9^	Pa
Yield stress (*σ*_y_)	100 × 10^6^	Pa
Poisson’s ratio (ν)	0.3	-
Density (*ρ*)	2700	kg/m^3^

**Table 5:** The results of VCW structural tests.

Degree	Maximum Stress (MPa)	Actual Maximum Displacement (m)	Desired Displacement (m)
0	5.3	0.005	0.005
2	22.5	0.023	0.022
4	35.2	0.033	0.033
6	50.9	0.047	0.046
8	69.9	0.063	0.062
10	81.5	0.073	0.07

## Data Availability

Data are contained within the article.
